# Point‐of‐care ultrasound use for vascular access assessment and cannulation in hemodialysis: A scoping review

**DOI:** 10.1111/sdi.12909

**Published:** 2020-08-03

**Authors:** Monica Schoch, Paul N. Bennett, Judy Currey, Alison M. Hutchinson

**Affiliations:** ^1^ School of Nursing and Midwifery Deakin University, Geelong Geelong Vic. Australia; ^2^ Medical & Clinical Affairs Satellite Healthcare San Jose CA USA; ^3^ School of Nursing and Midwifery Centre for Quality and Patient Safety Research Institute for Health Transformation Deakin University, Geelong Geelong Vic. Australia; ^4^ Monash Health Clayton Australia

## Abstract

Point‐of‐care ultrasound (POCUS) for access assessment and guided cannulation has become more common in hemodialysis units. The aims of this scoping review were to determine: circumstances in which renal nurses and technicians use POCUS; the barriers and facilitators; and evidence of the effects of POCUS in guiding assessment and cannulation. A search was conducted of CINAHL, Medline, Cochrane Database of Systematic Reviews, Cochrane Central Register of Controlled Trials, and ProQuest, Trove and Google Scholar as grey literature sources. Of 1904 publications, 21 studies met inclusion criteria (11 full text and 10 abstracts). These included primary research publications (n = 5), clinical observational cohort studies (n = 5), case studies (n = 3), published guidelines (n = 2), and published position papers (n = 6). POCUS was used for: assessing arteriovenous fistula (AVF) maturation; identifying landmarks and abnormalities; assessing alternate cannulation sites; performing new AVF cannulation; performing difficult cannulation; increasing cannulation accuracy; performing cannulation through stents; and patient self‐cannulation training. There were scant data on the barriers to, and facilitators of the use of POCUS, and a distinct lack of empirical evidence to support its use. These knowledge gaps highlight the need for further clinical studies, particularly randomized clinical trials, to test the effectiveness of POCUS in hemodialysis for assessment and guided cannulation.

## 1 INTRODUCTION

Vascular access is required to undertake hemodialysis treatment and can be attained via arteriovenous fistulae (AVF),^1^ arteriovenous grafts (AVG),^2^ or central venous access devices (CVAD).^3^ Cannulation of these vessels has traditionally used the “look, listen and feel” approach, known as “blind” cannulation.^4^, ^5^, ^6^ Unfortunately, complications of blind cannulation result in access damage, access failure, treatment delay and increased requirement for CVADs.^7^, ^8^, ^9^, ^10^, ^11^ Furthermore, the common practice of repeated cannulation of an AVF/AVG in one location weakens the vessel wall, increasing the risk of aneurysms.^12^ Over time, due to turbulent blood flow within the aneurysm, intra‐aneurysmal thromboses can form and the skin can become weaker and shinier, affecting cannulation, and prolonging bleeding times post dialysis.^13^

To avoid blind cannulation, some clinicians have turned to ultrasound technology to visualize vessels. This practice shift has emerged from a history of sonographers using large ultrasound machines to determine the vessels available for surgical creation of an AVF and to diagnose access complications.^14^, ^15^ In the early 2000s, portable ultrasounds allowed non‐sonographer clinicians to use ultrasound at point‐of‐care to assess accesses prior to cannulation, and guide cannulation with real time vision.^6^, ^16^, ^17^ However, despite ultrasound devices becoming smaller, more affordable and increasingly available, hemodialysis clinical uptake remains low.^18^

Ultrasound for assessment and guidance is a useful adjunct to other vascular access clinical assessments, particularly for vessels that are new, small, mobile, or tortuous.^5^, ^6^ Currently, the use of ultrasound for assessment and cannulation guidance is recommended in only one hemodialysis guideline worldwide; however, this recommendation is not supported by empirical evidence.^19^ Furthermore, it is not clear what evidence currently exists in relation to ultrasound use for vascular access assessment and cannulation guidance.

Scoping reviews are generally conducted when there is a distinct lack of randomized controlled trials in a particular clinical research area that precludes synthesis of findings from homogenous data sets to undertake a systematic review and meta‐analyses.^20^ A scoping review allows for the inclusion of published and non‐published material, and includes any heterogeneous data to provide an overview of the breadth (or lack) of information available on a particular topic of interest.^21^, ^22^, ^23^ The purpose of this scoping review was to investigate current available literature and gaps in evidence related to point‐of‐care ultrasound (POCUS) for hemodialysis vascular access, and particularly POCUS‐guided cannulation in hemodialysis vascular access. Information gathered from this review will inform requirements for clinical practice and further clinical research.

## 2 AIM

The aim of this scoping review was to answer the following questions:

1. In what circumstances do renal nurses and technicians in hemodialysis units use POCUS for cannulation guidance of vascular access?
2. What are the reported barriers and facilitators related to the experience of renal nurses and technicians using POCUS for cannulation guidance in hemodialysis?
3. What is the empirical evidence to support the use of POCUS‐guided cannulation of vascular access in hemodialysis?

## 3 METHODS

The scoping review was guided by a predefined protocol informed by contemporary methodologies for scoping reviews, specifically following the Preferred Reporting Items for Systematic Reviews and Meta‐Analyses‐Extension for Scoping Reviews reporting guidelines.^20^, ^22^, ^24^

### 3.1 Eligibility criteria

The lack of evidence for use of POCUS‐guided cannulation in hemodialysis informed the decision to include grey literature such as conference abstracts, literature reviews, opinion pieces, position papers, letters, and theses, along with the published peer‐reviewed primary research studies. The date range for retrieval was 1980 onwards because ultrasound was not used in hemodialysis prior to the 1980s. Inclusion criteria were literature reporting: adult (18+ years) hemodialysis patients; hemodialysis patients with AVF; hemodialysis patients with AVG; use of POCUS for cannulation by nurses and renal technicians; use of POCUS for assessment by nurses and renal technicians; use of POCUS for AVF monitoring; and studies based in any hemodialysis setting (in‐center, satellite, home).

Publications were excluded if they: were not published in English; investigated POCUS‐guided peripheral cannula insertion or POCUS use in general vascular access; included iatrogenic AVF; were animal studies; referred to guided ultrasound being undertaken by a sonographer, radiographer, physician or surgeon; investigated CVAD insertion; investigated ultrasound dilutional access flow monitoring; or examined cannulation techniques but did not mention use of ultrasound. While there are frequently cited abstracts,^25^, ^26^ letters^27^, ^28^ and other published works related to the use of POCUS in hemodialysis by nephrologists,^17^, ^29^ surgeons,^30^ or sonographers,^31^ these did not meet agreed inclusion criteria, so were excluded from this review.

### 3.2 Database search

A bibliographic database search of CINAHL complete, Medline complete and EMBASE was conducted. Restricted to the years 1980‐2019, MeSH (Medical Subject Heading) and CINAHL terms were searched for in both title and abstract. Search terms included: “hemodialysis,” “vascular access,” “AVF,” “AVG,” “ultrasound,” “sonography,” “cannulation,” “miscannulation,” and “nurse”. Search terms were combined using Boolean operators AND and OR. The Cochrane Database of Systematic Reviews and Cochrane Central Register of Controlled Trials were searched without year restrictions. Sources of grey literature searched included ProQuest, Trove, and Google Scholar.

Publication titles and abstracts were independently reviewed by two reviewers (MS, PB, or MS, AH) using the Rayyan QCRI system.^32^ Reviewers resolved all conflicts without the requirement of a third reviewer.

### 3.3 Data extraction

Data extraction was undertaken by one researcher (MS) using a tool based on the inclusion criteria and checked by a second researcher (AH) with consensus reached on relevant data. Data extraction tables were devised by the authors to collate and present information according to the scoping review questions (see Tables 1 and 2).

**TABLE 1:** Data extracted from included clinical research publications

First author	Study design	Sample	Inclusion/exclusion	Types of AVF studied	Ultrasound use	Type of ultrasound	Duration of study	Variables measured	Outcomes
Adams, B^43^ 2015 United States	Case study	3 HD patients	I: AVF unable to achieve 3 consecutive successful cannulations E: not stated	Radiocephalic Brachiocephalic Brachiobasilic	Assessment Real‐time guidance	Sonic Window©, Coronal Mode Ultrasound Device (CMUD)	Unclear	Pre and post introduction of POCUS: Cannulation failure days Number of infiltrations, interventions and hospitalizations	**Mean cannulation failure** days = 190 d Infiltrations reduced from 7 to 0, interventions from 11 to 0 and hospitalizations from 6 to 0
Carneiro, F^38^ 2014 Brazil	Observational cohort study	157 HD patients 72.5% male. Mean age 65.9. 39% diabetes	I: Not stated E: Not stated	Not stated	Real‐time guidance	Doppler Ultrasound	10 y	Hematoma episodes >2 cannulation attempts	**Hematoma episodes**/ patient per year reduced from 2.63 to 1.71 (*P* = .054). **>2 cannulation episodes**/patient per year reduced from 4 to 1.78 (*P* = .0014).
Chua, J^39^ 2016 United Kingdom	Observational cohort study	56 HD patients	I: those with known cannulation difficulties, new AVF & revised AVF or AVG E: not stated	Not stated	Real‐time guidance	Not stated	5 y	Number of CVADs inserted before and after introduction of ultrasound	Patients no longer required temporary CVADs
Darbas Barbe, R^33^ 2016 Spain	Point prevalence audit	9 HD patients/area punctured AVFs. 44.5% male Mean age 73. 11% diabetes	I: Over 18, on HD, area puncture, without stenosis E: not stated	Brachiobasilic Brachio‐median cubital	Not stated	Doppler Ultrasound	1 mo	Depth and diameter of non ‐punctured section of AVF Arterial access flow Success of change to rope ladder	Seven patients could utilize longer vessel length therefore cannulation technique **changed from area puncture to rope ladder**
Farpour, F^40^ 2015 United States	Retrospective audit	17 HD patients	I: Initial cannulation difficult cannulation E: Not stated	Not stated	Real‐time guidance	Sonosite M‐turbo® ultrasound (Sonosite)	1 y	Post‐surgery cannulation time pre and post ultrasound introduction	**Successful cannulation** improved from 10.8 ± 1 to 7.1 ± 1 wk
Jian, L^44^ 2016 Australia	Case study	3 HD patients 66% male. Mean age 77 y	I: Stent grafts in situ in useable segment E: Not stated	Brachiocephalic Radiocephalic	Real‐time guidance	Not stated	Not clear	Stent separations Stent distortions Stent infections Stent fracture	**Stent separation** pre‐POCUS = 3, post POCUS = 0. **Stent distortion** pre‐POCUS = 1, post POCUS = 0 **Stent infections** pre‐POCUS = 1, post POCUS = 0 **Stent fracture** pre‐POCUS = 3, post POCUS = 0
Kumbar, L^34^ 2018 United States	Randomized prospective pilot study	9 HD patients (C:4, P:5) 60% male. Mean age 66. 60% diabetes	I: Newly created AVF E: not stated	C: Brachiobasilic U: Brachiobasilic Brachiocephalic	U: Assessment (29%) Real‐time guidance (61%)	Sonic Window©, (CMUD)	3 mo	Pre cannulation measurements And assessment time Cannulation attempts Reason for miscannulation Blood pump speed Patient pain score Patient comfort Cannulation time Infiltrations	**Pre‐cannulation assessment time and overall cannulation times were shorter in the control group** (*P* = <.001) **Cannulation attempts and infiltrations were** no different (*P* = .64) and (*P* = .93) **Blood pump speeds** were higher in ultrasound group (*P* = .04) **Patient pain score**(*P* = .12) **and comfort** were no different
Leung, P^45^ 2016 Australia	Case study	1 HD patient 77‐y‐old female Diabetes	Not Applicable	radiocephalic	Real‐time guidance	Not stated	Not stated	Cannulation success	Real‐time ultrasound detected false aneurysm and the danger of rupture was able to be addressed with surgery
Luehr, A^35^ 2018 United States	3‐phase Prospective Observational Cohort study	53 HD patients **Phase 1** 34 patients, **Phase 2A** 38 patients, 13 staff **Phase 2B**25 patients, **Phase 3** 17 patients (phases 2B and 3 18 staff)	I: Patients using AVF for HD E: Not stated	Not stated	Assessment Real‐time guidance	Not stated	13 mo	Missed Cannulations Cannulator experience versus miscannulations Age of AVF versus miscannulations	Phase 1 (pre POCUS purchase) = 15.5 **missed cannulations**/1000 cannulations versus Phase 3 (post POCUS policy and procedure implementation) = 4.9 missed cannulations/1000 cannulations >years **cannulator experience** correlated with <miscannulations Younger AVFs = more miscannulations
Marticorena, R^41^ 2014 Canada	Prospective cohort study	85 HD patients 59% male	I: New or complicated post procedure requiring needle repositioning E: Not stated	Not stated	Assessment Real‐time guidance	Not stated	10 wk	Miscannulations pre and post access procedure station (APS)	Miscannulations decreased from 125 miscannulations per 5 wk period to 7 (*P* = .0001)
Marticorena, R^36^ 2018 Canada	Prospective Observational Cohort Study	86 HD patients 58% male Mean age 65.3 68 nurses (1‐22 y' experience)	I: AVF or AVG uncomplicated blind cannulation E: infiltrations or required more than two needles	68 × upper arm 18 × lower arm (82 × AVF, 4 × AVG)	Assessment needle placement post cannulation	SonixTouch (Ultrasonix) OR Sonosite S‐Cath (Sonosite)	1 y	Needle position within first 30 min of dialysis after successful blind cannulation Access parameters (depth, diameter etc)	**Anterior wall position** = 53 (61.6%) **Posterior wall position** = 25(29.1%). Needles piercing the back wall were found in two patients **Centre of vessel** = 8 (9.3%) Association between **deep access** (>6 mm) and anterior needle position (*P* = <.001). There was no association with small **diameters** (< 6mm) and needle position (*P* = 1.0)
Paulson, W^42^ 2015 United States	Prospective observational cohort study	33 HD patients with AVF and AVG	I: Not stated E: Not stated	Not stated	Assessment Real‐time guidance	Sonic Window©, (CMUD) (Analogic corp.)	Not stated	Cannulation success	Staff successfully cannulated 33 AVFs and AVGs using POCUS
Wilson, B^37^ 2018 United States	Quantitative Descriptive Survey	252 Nurses and physician experts	I: Registered nurse and physician experts in vascular access in HD E: Not stated	Not applicable	Not stated	Not stated	Not stated	Variables that health practitioners consider as outcomes for successful cannulation	86.4% did not use POCUS‐ guided cannulation Cannulator skill with POCUS 30.4% (only 39.9% used POCUS in their practice)

Abbreviations: APS, access procedure station; AVF, arteriovenous fistula; AVG, arteriovenous graft; C, control group; CMUD, coronal mode ultrasound device; CVAD, central venous access device; E, excluded; HD, hemodialysis; I, included; P, POCUS group; HD,, hemodialysis; POCUS, point‐of‐care ultrasound.

**TABLE 2:** Data extracted from included guidelines and position papers

First author	Study design	Objective	Method	Recommendations	Conclusions
British Colombia Provincial Renal Agency^46^ 2017 Canada	Regional guideline	Provide guidelines for the use of POCUS in the care and management of vascular access in HD	Developed by BCPRA vascular access educators' group Reviewed by BCPRA renal educators' group (April 2017). Approved by BCPRA hemodialysis Committee (July 2017)	Consider POCUS for mapping of vessels and guiding cannulation Staff with appropriate training may utilize POCUS Training on the use of POCUS needs to include both theory and hands‐on practice	While no renal specific, evidence‐based guidelines regarding the use of POCUS exists, units that have implemented portable ultrasound report an improvement in vascular access related outcomes
Canadian Association of Nephrology Nurses and Technologists^19^ 2015 Canada	National guideline	Provide guidelines for care and management of all vascular access in HD	Developed 2006 by CEN & CHAC Updated by Vascular Access Guideline working group 2015: CHAC & CEN & CNNP	Use POCUS device to assess; diameter, depth, course, valves, narrowing and presence of thrombus POCUS for assessment and guided cannulation can optimize cannulation and needle placement	Improvement of cannulation skill is a requirement; therefore, a thorough assessment must be undertaken using POCUS, when available, to guide cannulation
Kamata, T^6^ 2016 Japan	Position paper	Describe POCUS‐guided cannulation theory and practical methods to promote further uptake	Review of literature and description of POCUS methods used	Preferred probe direction: transverse, however longitudinal is equally effective Optimal technique: one‐operator Sterile protection for probe is required POCUS can be used for initial cannulation, repositioning of needles, central venous access and femoral vein access Training ‐ 3 components: Theory, on the job/simulation training and reflection	POCUS use can minimize cannulation damage, resulting in better outcomes for patients with difficult access. Further research is required for POCUS to be considered as the standard care for difficult accesses
Marticorena, R^16^ 2015 Canada	Position paper	Outline competencies required for renal nurses to become proficient in use of POCUS in HD	Collaborative working group of vascular access nurses in Canada, developed competencies based on guidelines, expert opinion and experience	Training requires: Theory, practical on simulated models & patients. Three competency levels: 1. Basic, 2. Intermediate & 3. Advanced Expert clinicians validate levels of proficiency Approximately 500 POCUS‐guided cannulations to reach advanced level	Use of POCUS requires specialized training through theory and practice to provide the highest level of care to hemodialysis patients
Mills, L^47^ 2009 Canada	Position paper	Describe the adoption of POCUS into HD unit	Descriptive overview of implementation of POCUS to be presented	Successful cannulation reduces poor access outcomes Use POCUS for new AVF planning, buttonhole sites and difficult access	Not stated
Mills, L^48^ 2010 Canada	Position paper	Describe use of POCUS in HD unit.	Patient survey on perceptions of POCUS use, related to stress, infiltration frequency and overall experience	Implementation of POCUS is challenging Education via on the job training, written testing and certification	POCUS enhances nursing practice in hemodialysis
Schoch, M^11^ 2015 Australia	Position paper	POCUS ‐guided cannulation through a continuing professional development series	Description and review of POCUS	Required skills for renal nurse: Knowledge of POCUS basic physics POCUS identification of access abnormalities Awareness of barriers and facilitators to POCUS use	POCUS can have a positive effect on patient comfort, satisfaction and the lifespan of the access
Ward, F^5^ 2017 Canada	Position paper	Role of POCUS in cannulation, optimal practice/appropriate technique for POCUS‐guided cannulation	Literature review and opinion	Appropriate training by POCUS expert is required Simulated training is important POCUS can be used for AV mapping, measurements, assessment, technique choice and cannulation guidance	POCUS important for successful, safe, cannulation for difficult AV access, however blind cannulation is still an essential tool

Abbreviations: AV, arteriovenous; AVF, arteriovenous fistula; BCPRA, British Colombia Provincial Renal Agency; CEN, Greater Toronto Area Clinical Educators Network; CHAC, Canadian Hemodialysis Access Coordinators Network; CNNP, Canadian Nephrology Nurse Practitioners; HD, hemodialysis; POCUS, point‐of‐care ultrasound.

## 4 RESULTS

**FIGURE 1:**
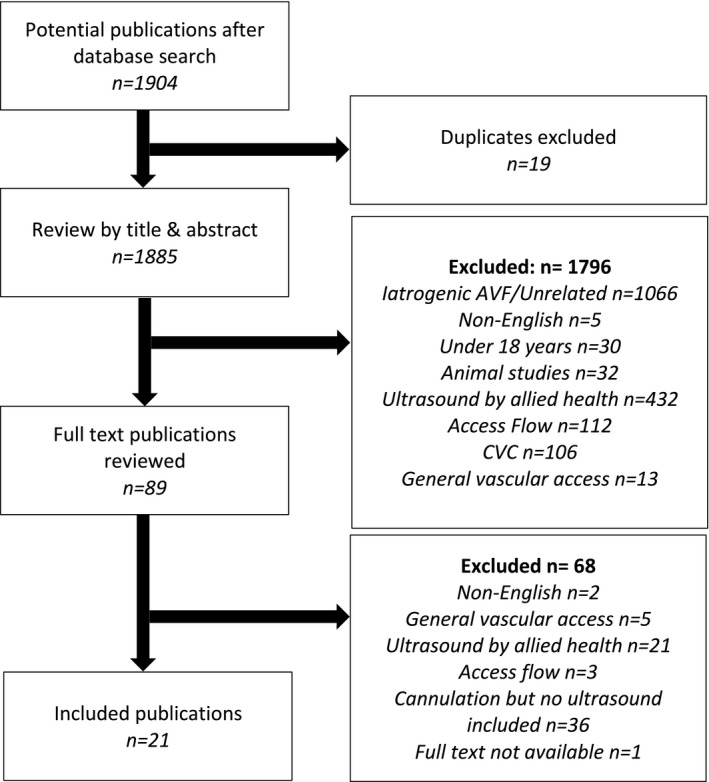
Preferred Reporting Items for Systematic Reviews and Meta‐Analyses flow diagram illustrating the screening process and results

A total of 1904 publications were retrieved, leaving 1885 unique records after duplicate removal. Screening of titles and abstract resulted in 89 publications requiring full text review. After full text review (MS/PB), another 68 publications were excluded because the authors referred to cannulation practices but did not mention POCUS guidance (n = 36), or POCUS was undertaken by allied health staff such as sonographers, physicians, or surgeons (n = 21). Three publications reported ultrasound use in relation to access flow rather than cannulation, two full text publications were not in English, five referred to general vascular access interventions, and one publication was unavailable in full text. Meeting the inclusion criteria were 21 publications from seven countries (Canada n = 8, United States of America n = 6, Australia n = 3, and one each from United Kingdom, Spain, Brazil, and Japan) (see Figure 1).

### 4.1 Characteristics of included publications

Characteristics of included publications are detailed in Tables 1 and 2. Of 21 publications (11 full text publications, 10 published abstracts), five were primary research publications,^33^, ^34^, ^35^, ^36^, ^37^ five clinical observational cohort studies (abstracts only),^38^, ^39^, ^40^, ^41^, ^42^ three case studies (abstracts only),^43^, ^44^, ^45^ two published guidelines,^19^, ^46^ four published position paper publications,^5^, ^6^, ^11^, ^16^ and two position papers were reported as abstracts.^47^, ^48^ Fifteen studies provided information regarding the context in which POCUS was used by nurses and technicians in hemodialysis units. These contexts included: assessing new AVF maturation (three publications—two were abstracts only)^5^, ^40^, ^42^; identifying landmarks and abnormalities (five publications—two were abstracts only)^5^, ^6^, ^11^, ^38^, ^45^; assessing for alternate cannulation sites (two publications—one was abstract only)^33^, ^42^; new AVF cannulation (four publications—one was abstract only)^5^, ^34^, ^39^, ^40^; difficult access cannulation (eight publications—four were abstracts only)^5^, ^6^, ^34^, ^35^, ^40^, ^41^, ^43^, ^47^; increasing cannulation accuracy (two publications)^34^, ^36^; successful cannulation through stents (one publication—abstract only)^44^ and patient self‐cannulation training (one publication).^5^ Six publications, one of which was an abstract only, identified some possible barriers to and facilitators of the use of POCUS in the hemodialysis setting,^5^, ^6^, ^16^, ^37^, ^42^ and six publications detailed guidelines or procedural requirements to undertake POCUS by renal nurses and technicians.^5^, ^6^, ^11^, ^16^, ^19^, ^46^

### 4.2 Context requirements for POCUS

In 15 publications, the actual and potential use of POCUS by renal nurses and technicians in hemodialysis units was described. Specifically, assessment of the maturation of a new AVF, identifying landmarks and abnormalities, assessing for alternate cannulation sites, cannulating new AVF, cannulation difficulties, increasing cannulation accuracy, cannulating through stents, and patient self‐cannulation issues were addressed.

#### 4.2.1 Assessing new AVF maturation

Three publications reported use of POCUS to assess new AVF maturation.^36^, ^40^, ^42^ POCUS was successfully used to assess and identify AVFs that had not reached the minimum required diameter of 6mm to enable cannulation.^40^ Based on their early experience, Paulson et al^42^ claimed that POCUS had the potential to be used more consistently to measure changes in AVF luminal diameter. However, these findings were only reported briefly in abstracts, and the authors provided no detailed data to support their claims. In a Canadian prospective cohort study, an association between deep access (>6 mm from skin surface) and anterior needle position (resting at the top of the vessel lumen) was found.^36^ This study demonstrated no association between small diameter and needle position, indicating that AVFs with smaller diameters can be cannulated successfully.^36^ Due to the current lack of evidence, Ward et al^5^ also recommended further research into the use of POCUS assessment of AVF maturation.

#### 4.2.2 Identifying landmarks and abnormalities

Point‐of‐care ultrasound can be used to view the vessel if abnormalities are detected on physical assessment^38^ and allows for the creation of a visual map of the AVF to identify straight and curved sections.^5^ Schoch et al^11^ argues that POCUS can also be used as an adjunct to physical assessment, complementing the skillset of the cannulator. Additionally, POCUS can be useful in identifying valves,^11^ pseudoaneurysms,^11^, ^45^ aneurysms,^11^ adjacent artery or nerves^5^, ^6^ or hematomas,^5^, ^11^ in order to improve cannulation.^5^ Anecdotal reports suggest that cannulation‐induced intraluminal thrombus does not require diagnostic imaging by a sonographer, and if the area is left for a period of weeks the clot will dissipate allowing for recannulation of that area without incident.^11^ An example of identifying abnormalities with POCUS was detailed in an Australian case study of a 77‐year‐old hemodialysis patient who had a radial artery pseudoaneurysm identified when POCUS was used for guided cannulation of the overlying AVF. The pseudoaneurysm resulted from cannulation infiltration through the AVF during blind cannulation.^45^ Therefore, POCUS shows promise as a tool, not only to assist in identifying abnormalities within the vessels, but also to prevent harm to patients from blind cannulation extravasation.^45^

#### 4.2.3 Assessing for alternate cannulation sites

The use of POCUS to assess for alternate cannulation sites was reported in two publications.^33^, ^42^ A point prevalence audit study undertaken in Brazil identified that 23% of their AVFs were area punctured. However, following the use of POCUS to assess vessel parameters for more viable length, 78% of the area puncture cases were successfully transitioned to the recommended rope ladder cannulation technique (the remainder required surgical intervention).^33^ Following a prospective observational cohort study, reported in an abstract only, Paulson et al^42^ concluded POCUS had the potential to identify alternate cannulation sites to avoid overuse of certain areas.

#### 4.2.4 New AVF cannulation

One randomized prospective pilot study was undertaken specifically to investigate new AVFs requiring cannulation due to the higher needle infiltration risk in this cohort.^34^ This small cohort study (POCUS n = 5, blind cannulation n = 4) did not show a difference between infiltrations in the POCUS group versus the blind cannulation group. However, Kumbar et al^34^ did report patient satisfaction was higher in the POCUS group, but the time taken to assess vessels using POCUS and time taken to cannulate using POCUS were longer.^34^ Authors of a retrospective audit reported that when POCUS was used to guide cannulation, time to cannulation success (from date of surgery) reduced from 10.8 ± 1 weeks (n = 18) in the previous 12 months to 7.1 ± 1 (n = 17) weeks in the study period.^40^ In a position paper, Ward et al^5^ advised successful cannulation of new AVFs is critical to provide adequate dialysis as soon as required and to improve the experiences of patients related to early access miscannulations. Similarly, Chua^39^ proposed that the more accurate the early cannulation, the less likely it is the patient will require CVAD. In sum, POCUS has the potential to improve the cannulation experiences for patients with new AVFs.^5^, ^34^, ^39^

#### 4.2.5 Difficult access cannulation

The increasing prevalence of co‐morbidities and an ageing population has resulted in increasingly complex access cannulation.^5^, ^6^ Definitions of “difficult access” vary and include: the inability to achieve three successful dialysis sessions (two needles in each), with resulting CVAD use^43^ or the requirement of more than two needles in a session more than once per week.^41^ Others have referred to difficult access in terms of length, diameter, location, depth or tortuosity.^5^, ^34^, ^47^ In practice, Farpour et al^40^ found that the use of POCUS guidance for cannulation was successful when five “difficult access” cases were accurately cannulated, when blind cannulation had previously failed. POCUS for guided cannulation was reported to enable clear visualization of the vessel and therefore has the potential to decrease cannulation mistakes^34^ and consequences such as infiltrations,^41^ thus increasing the cannulation success rate.^5^, ^47^

Adams et al^43^ reported difficulties in cannulating patients with AVF tortuosity, multiple vessel infiltrations and comorbidities of diabetes and obesity, thus lengthening the time CVADs remained in situ. In one 3‐phase prospective cohort study, prior to the introduction of POCUS assessment and guidance, patients with diabetes had 22/1000 miscannulations; however, this decreased to 1.3/1000 with the introduction of POCUS assessment and guidance.^35^ Similarly, a decrease in the requirement for more than two cannulations, from four episodes per patient year to 1.78 episodes per patient year has been reported.^38^ Adams et al^43^ presented a case study reporting that prior to the introduction of POCUS into the hemodialysis unit, three patients with difficult access (defined as unable to successfully cannulate on three consecutive occasions) had a number of instances of infiltrations (n = 7), interventions (n = 11) and hospitalizations (n = 6). Then in the post‐POCUS implementation period (pre and post timeframes undisclosed) none of these events were recorded for the three patients.

Authors of a Canadian prospective cohort study reported implementing an access procedure station with POCUS in an attempt to decrease adverse cannulation events.^41^ Marticorena et al^41^ found a reduction in adverse cannulation events (extravasation, miscannulation, venous spasm) from 125 events to seven events over two 5‐week periods. Findings from Paulson et al's^42^ prospective cohort study, presented in abstract form only, indicated that POCUS represented a significant improvement in technology for managing and cannulating ‘difficult’ accesses.^42^

#### 4.2.6 Increasing cannulation accuracy

Increasing cannulation accuracy is vital to ensure correct needle position and successful access. Results from a prospective cohort study identifying intraluminal needle position showed that after blind cannulation, venous needle position was assessed using POCUS (n = 86) in the first 30 minutes of hemodialysis, only 9.3% of venous needles were located with the tip pointing into the centre of the vessel.^36^ Marticorena et al^36^ suggested that to minimize the possibility of mechanical trauma the needle should be located in the center of the vessel. It was noted that arterial needle location could not be assessed due to the close proximity of the needle tip to the hub of the venous needle and the tapes securing it (POCUS cannot penetrate securement tapes).^36^ There was a positive correlation between deeper vessels (>0.6 mm from the skin surface) and needles lying against the top of the vessel lumen, possibly due to securing the hub with tape after insertion to prevent dislodgement.^36^ Four needles were located with tips piercing the vessel lumen with no apparent discomfort to the patients, nor changes in machine pressures to indicate an issue with needle position.^36^ Staff repositioned these needles under POCUS guidance to decrease possible damage to the vessel lumen during hemodialysis.^36^ In a randomized prospective pilot study, Kumbar et al^34^ found that dialysis machine pump speeds in the POCUS group could be set higher, without any increase in dialysis machine pressures (313.2 ± 73.7 mL/min vs 264.2 ± 60.1 mL/min); possibly due to correct positioning of needles in vessels.

#### 4.2.7 Successful cannulation through stents

Blind cannulation through stents surgically inserted into the useable segment of AVFs is rare and can challenge clinicians. Adverse outcomes reported by Jian et al^44^ in a case study of three patients (reported as an abstract) were: stent separation from the vessel wall, stent fracture or distortion, infection or pseudoaneurysm. Arising from this study was a recommendation for POCUS assessment and guided cannulation to be used to prevent stent separation and damage by visualizing the needle tip insertion into stents in the useable segment of the AVF.^44^ POCUS may prevent issues with the needle sliding between the stent and the lumen causing stent separation.^44^ Jian et al^44^ suggest POCUS has the potential to decrease vessel damage and stent damage from misdirections and miscannulations.

#### 4.2.8 Patient self‐cannulation training

Only Ward et al,^5^ in a position paper, proposed that POCUS could be a useful tool when training patients to self‐cannulate for home hemodialysis. POCUS was claimed to have the potential to give patients a sense of the size, depth and direction of the vessel, thus decreasing possible damage to the back wall of the vessel during cannulation.^5^

### 4.3 Barriers and facilitators to renal nurses' and technicians' POCUS use

There was limited literature related to barriers to and facilitators of the use of POCUS for assessment and guidance in hemodialysis. Barriers identified in the literature included: the extra time required to use POCUS for assessment or guidance,^5^ limited availability of devices,^6^ reluctance to use new technologies^42^ and that POCUS could have a significant impact on workflow in already busy hemodialysis units.^5^, ^34^ The following highlights the limited evidence related to barriers to using POCUS. Time measurements recorded by Kumbar et al^34^ indicated an increase in the length of time for POCUS‐guided cannulation (from skin preparation to needle in place) (41.1 ± 70.6 seconds) compared to blind cannulation (25 ± 27.9 seconds). Paulson et al^42^ suggested that the use of POCUS guidance, in their experience, generally adds an extra 1‐3 minutes to cannulation time.

In an online survey exploring renal healthcare worker perceptions of cannulation outcomes, only 13.6% (n = 34) of 252 respondents reported they used POCUS for guided cannulation.^37^ Respondents, 75% of whom were registered nurses, had between 7 months and 44 years' experience in nephrology (*M* = 20 years). Their survey responses highlighted that only 30.4% of respondents felt cannulator skill with POCUS‐guided cannulation was important for cannulation success.^37^ Alternatively, 97.6% of respondents felt that cannulators needed to have the ability to assess the AVF and 96% agreed that the level of cannulators' knowledge of the AVF anatomy is important to successful cannulation.^37^

Anecdotal reports indicated appropriate training and competency testing increased nurses' abilities with using POCUS for assessment and guidance.^16^, ^39^ According to Wilson et al,^37^ more in‐depth understanding of the perceptions of nursing and technical staff, and patients, regarding the possible barriers to and facilitators of POCUS use is required.^37^ Overall, the authors concluded that POCUS could be a factor in improving cannulation outcomes and recommended further research into nurses' perceptions about the use of POCUS in hemodialysis.^37^

### 4.4 Procedural Requirements for POCUS

Two hemodialysis vascular access guidelines from Canada recommended POCUS for assessment and/or cannulation.^19^, ^46^ Recommendations from the Canadian Association of Nephrology Nurses and Technologists (CANNT)^19^ for use of POCUS by hemodialysis nurses are based on expert opinion. The regional British Colombia Provincial Renal Agency^46^ guideline recommendations are based on the CANNT^19^ document and anecdotal experiences documented in various discussion publications from units around the world.^6^, ^16^, ^18^, ^41^ Both sets of guidelines recommended the use of available POCUS for: assessment of the vessels prior to cannulation including vessel diameter, depth, presence of valves, stenosis and thrombosis; real time guided cannulation to optimize needle placement; and assessment of needle position and possible realignment in the vessel.^19^, ^46^

More recently, the Kidney Disease Outcomes Quality Initiative in the United States has released updated vascular access guidelines.^49^ Based on expert opinion, these guidelines state it “is reasonable to use ultrasound to help determine direction of flow and proper needle placement in the AV access of select patients as needed and performed by trained operators, to prevent cannulation complications.”^49^

Four publications^5^, ^6^, ^11^, ^16^ indicated the need for staff training to undertake POCUS assessment and guided cannulation. However, only three publications included detailed training requirements to achieve competence in POCUS‐guided cannulation.^5^, ^6^, ^16^ This training included didactic teaching of the theoretical principles of ultrasound, hands‐on training by expert POCUS users using simulated models,^5^, ^6^, ^16^ approximately 10 supervised POCUS‐guided cannulations prior to independent use (based on CVAD POCUS‐guided insertion guidelines)^5^ or 10 supervised POCUS‐guided cannulations on simulation models, three supervised POCUS‐guided cannulations on patients,^16^ and reflection after training.^6^ To be able to reach expert status in POCUS‐guided cannulation, Marticorena et al^16^ suggested approximately 500 guided cannulations are required.

The integration of POCUS into local cannulation guidelines,^47^ and the facilitation of training, written tests and certification after six successful cannulations using ultrasound were described in two position paper abstracts.^48^ As these abstracts were descriptive and not research‐based with methods and results reported, there is scant detail about how the education program was operationalized.

General steps required to achieve successful POCUS‐guided cannulation were outlined in two publications,^5^, ^6^ with one taking a step further by scaffolding competencies into basic, intermediate and advanced levels.^16^ The authors of two position papers specified from their experience that the preferred ultrasound probe direction is transverse as the first preference, and longitudinal the second.^5^, ^6^ Ward et al^5^ and Kamata et al^6^ recognized, however, that either transverse or longitudinal are options, and in light of the lack of empirical evidence to support either, personal preference is appropriate. The steps for POCUS‐guided cannulation as recommended^5^, ^6^, ^11^, ^16^ are summarized in Table 3 to highlight current recommended practice.

**TABLE 3:** Current recommended steps for POCUS‐guided cannulation ^5^, ^6^, ^11^, ^16^

Practice step	First author			
	Ward^5^	Kamata^6^	Schoch^11^	Marticorena^16^
Complete physical assessment of the AVF				X
Use sterile probe cover and sterile gel in individual sachets	X	X	X	X
Observe and evaluate the vessel	X	X		X
Measure diameter and depth using the ‘freeze’ and calliper functions	X			X
Set depth to minimum to see vessel in middle of screen	X			
Sterilize the skin	X	X		
Administer local anesthetic prn	X	X		
Apply tourniquet	X	X		
Not too much pressure on the probe	X			
Slide, rotate, compress, tilt and angle probe for best assessment				X
Identify artifact, reverberation, enhancement and acoustic shadowing			X	X
Identify presence of valves, pseudoaneurysm, aneurysm, hematoma, back walling/coring			X	
Orient the needle guide	X			
Insert the needle	X	X		
Move the probe to visualize the shaft	X			
If the needle cannot be seen do not advance, back up and redirect	X	X		
Advance needle	X			
Tape securely	X			

Abbreviation: AVF, arteriovenous fistula.

According to Ward et al,^5^ although POCUS guidance can assist in managing difficult cannulations in AVFs, it is not feasible or necessary to use POCUS guided cannulation on all AVFs. Additionally, Ward et al^5^ argued POCUS has a role as an adjunct tool for cannulating new and difficult to access AVFs. Robust training and competency completion were considered by Marticorena et al^16^ as essential for those undertaking assessment and/or guidance using POCUS. The advantages of using POCUS for assessment and guidance based on the anecdotal clinical experience of Schoch et al^11^ are; allowing clinicians to visualize the interior of the vessels; avoiding miscannulations; and avoiding expensive tests or interventions.

## 5 DISCUSSION

This scoping review identified 11 full text publications published between 2015 and 2018, and 10 abstracts from conference proceedings published between 2009 and 2016, that related to the use of POCUS for assessment and guidance for hemodialysis vascular access by nurses or renal technicians.

Findings from this scoping review showed some positive outcomes for patients when POCUS is used for assessment and guidance by renal nurses and technicians. Research publications reported POCUS has the potential to: reduce cannulation complications such as miscannulation, misalignment, and extravasation of the vessel; detect vessel wall needle infiltration in the absence of signs or symptoms; identify abnormalities not visible from skin surface; and detect other areas of usable vessel to decrease area puncture cannulation.^33^, ^34^, ^35^, ^36^, ^37^ However, extra time was taken to complete POCUS assessment and/or POCUS‐guided cannulation.^34^ These studies did provide methodological insights to assist the design of future studies to empirically support or refute the effectiveness of POCUS use in hemodialysis settings. Insights such as approximate numbers required to recruit, experienced operators needed, randomization of participants and possible phases of data collection.

Two recent scoping reviews on the topic of cannulation in hemodialysis^8^, ^50^ in general were reviewed. Jaensch et al^50^ concluded that cannulation complications are a common problem, rope ladder cannulation technique should be enforced, and POCUS can be a useful tool for combatting difficult cannulations. Harwood et al^8^ suggested there is no current consensus in the literature on what constitutes “successful cannulation,” and a lack of emphasis on patient perceptions of cannulation along with associated pain and anxiety levels of patients. The latter authors noted POCUS skill across nurses was inconsistent and there is a distinct lack of nursing research on this topic.

Recently, Niyyar^51^ published details regarding a POCUS education workshop (run by a nephrologist) for POCUS guidance by renal nurses and technicians in hemodialysis access, similar to the proposed education focus of the position papers included in this scoping review.^5^, ^6^, ^11^, ^16^ Niyyar's^51^ workshop included a didactic teaching component and a hands‐on component with simulated models. Niyyar^51^ found that POCUS workshops have the potential to empower the staff and increase the confidence using POCUS guidance for cannulation. This was evidenced by the fact none of the participants felt “extremely confident” prior to the workshop but 36.1% of participants were “extremely confident” with POCUS, following completion of the workshop.^51^

### 5.1 Limitations of the literature

There were limitations with some of the publications included in this scoping review. Notably, 10 abstracts from conference proceedings were included, no follow‐up full text publications for these abstracts could be sourced. In some studies, study design or methods were unclear, such as not detailing randomization processes. Other limitations were: results having no statistical significance and insufficient power due to low participant numbers; inconsistent time frames across phases within and between studies; information collected from the electronic medical record, thus relying on accuracy and completeness of nurses' and other clinicians' documentation of events; uneven crossover of participants from one group to anther; inconsistent use of POCUS guidance versus POCUS assessment in intervention groups; studies designed to only visualize one needle, not both needles in POCUS; low response rates to surveys; and poorly operationalized concepts or omitted methodological definitions of terms like “expert.” None of the included publications outlined costs associated with the use of POCUS in hemodialysis units.

### 5.2 Limitations of the scoping review

This scoping review has several limitations. First, any studies that were not published in English were excluded, thus possibly omitting relevant information from non‐English publications. We also excluded studies relating to use of POCUS by allied health professionals, this may have also excluded information that, whilst not directly related, could be applied by renal nurses and technicians in practice.

### 5.3 Empirical evidence

There is a lack of published evidence related to the use of POCUS in hemodialysis vascular access, particularly related to POCUS guidance during cannulation,^35^ and there is a dearth of empirical evidence to advise on POCUS use.^5^ Adequately powered randomized controlled studies are required to determine whether POCUS may lead to improvements in vascular access outcomes.^34^ Prospective clinical trials are required with a specific focus on the indications for use of POCUS, comparison with the standard practice of blind cannulation, and measurement of outcomes related to the dependence on CVAD and optimal POCUS techniques.^5^ New guidelines call for “rigorous study of use of ultrasound‐guided cannulation—its safety, efficacy, and impact in busy dialysis units.”^49^

### 5.4 Implications for clinical practice

The small number of clinical cohort publications and position papers and conference proceedings suggest positive outcomes are associated with the use of POCUS for hemodialysis access for clinical use. The findings of the included research show promise, particularly in relation to identifying possible access abnormalities^40^, ^45^ (such as pseudoaneurysms, presence of clot, tortuosity, and stenoses), facilitating routine and difficult cannulations^40^, ^42^ (which may decrease area puncture and aneurysm formation) and decreasing miscannulations and needle manipulation, thus minimizing access damage^6^, ^11^, ^35^, ^38^, ^39^, ^43^ (such as back wall damage and hematoma formation from infiltrations). However, these findings need to be treated with caution given the limitations in the existing evidence base.

### 5.5 Implications for further research

There is a need for further clinical studies into the use of POCUS for assessment and guided cannulation. In particular, randomized clinical studies to test the effectiveness of this intervention on key patient outcomes are recommended, with recognition that recruiting large sample sizes will be required to adequately power such studies. To assist with future meta‐analyses, common sets of variables and standardized measures are recommended. Inclusion of the following variables and design characteristics is suggested. Variable to include: the age, depth and diameter of AVFs, the time taken to conduct cannulation, the number of miscannulations and CVAD line days, intraluminal needle position, patient pain scores, dialysis machine pump speed and AVF access flow. Design characteristics to include: robust demographic information (including comorbidities that can affect vessel quality), consistent cannulators (where practical), record of cannulation and ultrasound technique (including probe direction), type of ultrasound device used, nurse experience in cannulation and POCUS, nurse training in cannulation POCUS and an even range of different types of AVF (where possible). A cost benefit analysis related to the cost of POCUS machines and additional consumables and the benefits (including cost savings) of POCUS is also important to include in future research. Research to address implementation of POCUS‐guided cannulation is needed to understand patient and staff perceptions and their satisfaction with POCUS assessment and guidance. Perceived barriers to and facilitators of the use of POCUS in hemodialysis units is also required.

## 6 CONCLUSIONS

This scoping review has highlighted that whilst there are positive reports on the use of POCUS assessment and guided cannulation in hemodialysis, there is a distinct lack of robust studies evaluating POCUS in this context. In addition, gaps in our knowledge regarding staff perceptions and perceived barriers to, and facilitators of, POCUS use exist. Recommendations based on expert opinion suggest that use of POCUS for assessment and cannulation guidance has the potential to provide improved outcomes for patients' AVF. Further research into these possible outcomes is required in order to substantiate or refute the published opinions of experts and provide higher quality evidence and more precise guidance for practice.

## CONFLICT OF INTEREST

No gifts of material, grants, sources of support or potential conflicts of interest.
